# Sudden death due to cardiac contusion: Forensic implications in a rare pediatric case

**DOI:** 10.1111/1556-4029.14741

**Published:** 2021-05-07

**Authors:** Guendalina Gentile, Stefano Tambuzzi, Giulio Giovanetti, Riccardo Zoja

**Affiliations:** ^1^ Dipartimento di Scienze Biomediche per la Salute Sezione di Medicina Legale e delle Assicurazioni Università degli Studi di Milano Milano Italy

**Keywords:** autopsy, blunt chest trauma, cardiac contusion, cardiac rupture, forensic pathology, pediatric, sudden death

## Abstract

Blunt chest trauma (BCT) often results in blunt cardiac injuries of little clinical concern, but cases of severe heart damage with high mortality rates have also been described. In particular, BCT should never be underestimated, especially when it is located in the anterior thoracic region. Among traffic accidents, motorcyclists are the most vulnerable and at the greatest risk. We report the case of a 14‐year‐old boy who experienced BCT following a motorcycle accident. He was evaluated at the hospital and was found to be in good medical condition, without bruises or rib fractures. Electrocardiography revealed a left bundle branch block. The patient was kept overnight for observation and was discharged the following morning in a good health condition. However, five days later, the patient suddenly died. Autopsy revealed a cardiac contusion associated with a full‐thickness myocardial rupture and massive hemopericardium. Histologically, hemorrhagic infiltration foci, fibrin deposits, neutrophilic granulocytes, and well‐defined areas of necrosis were detected in the context of recent fibrosis. Coronary thrombosis was not observed. The cause of death was identified as cardiac contusion that caused myocardial necrosis and, ultimately, cardiac rupture. Because the boy suffered a recent BCT and was assessed at the hospital, issues of medical malpractice were raised. This case demonstrates the potential lethality of blunt chest trauma in pediatric patients and demonstrates the importance of not underestimating such events, even in the absence of clinically identified chest injuries.

**Highlights**

- Pediatric blunt chest trauma may cause potentially lethal cardiac contusions.
- Even in the absence of clinically identifiable chest injuries, cardiac contusions may be present.
- Cardiac contusions may cause myocardial necrosis and full‐thickness heart rupture

## 1 INTRODUCTION

The heart is relatively well protected within the thoracic cavity and is located at the center of the chest, with the lungs on either side, the sternum anteriorly, and the vertebral column posteriorly. However, it is vulnerable to penetrating and blunt trauma (blunt chest trauma, BCT), which may result in blunt cardiac injury (BCI) [1]. Penetrating injuries are considered very severe and generally require urgent medical treatment. However, blunt chest trauma should not be underestimated, especially if located in the anterior thoracic region, which is associated with high mortality rates [2]. Approximately 64% of all BCIs occur as a consequence of falls from a height [3], blasts, some high‐risk sports (i.e., water skiing, rugby, and basketball), assaults [4], and road traffic collisions [5]. The latter accounts for at least 20% of all deaths due to post‐traumatic cardiac rupture and pericardial tamponade [6]. Moreover, among road accidents, complex mechanisms of exposure and speed make the subcategory of motorcyclists particularly vulnerable to BCI with a high risk of death [6]. One of the main causes of death in such cases is traumatic rupture of the aortic isthmus [7].

In this report, we present a forensic case of blunt chest trauma that occurred in a 14‐year‐old boy following a motor vehicle accident. A few days after the trauma, the boy suddenly died due to heart rupture with pericardial tamponade. This case is notable as it shows the potential lethality of BCT, which requires proper clinical investigations aimed at excluding an underlying occult and potentially fatal heart injury.

## 2 THE CASE

A 14‐year‐old boy was involved in a road accident while driving his moped and wearing a helmet; in an attempt to avoid a car approaching from the opposite direction, he lost control of his motor vehicle, fell to the ground, and hit his chest against one of the speed twist throttles. The patient was transported to the hospital for examination. He appeared to be in a good health condition, complaining exclusively of widespread thoracic pain on palpation. No bruises were observed, and X‐ray examination of the chest excluded the presence of rib fractures. All other anatomical areas were undamaged. His blood count was normal, and an electrocardiogram (ECG) was performed, which revealed a normal heart rate and a left bundle branch block (LBBB). The patient was kept overnight for observation, was discharged the next morning in a healthy condition, and was invited to return the following week for a further cardiological check‐up. However, five days later, he suddenly collapsed and died immediately before the arrival of the rescue team. They performed resuscitation maneuvers on the way to the nearest hospital, where the death was declared. To clarify the cause of death, the investigating magistrate ordered a judicial autopsy that took place two days later.

### 2.1 Gross examination

**FIGURE 1:**
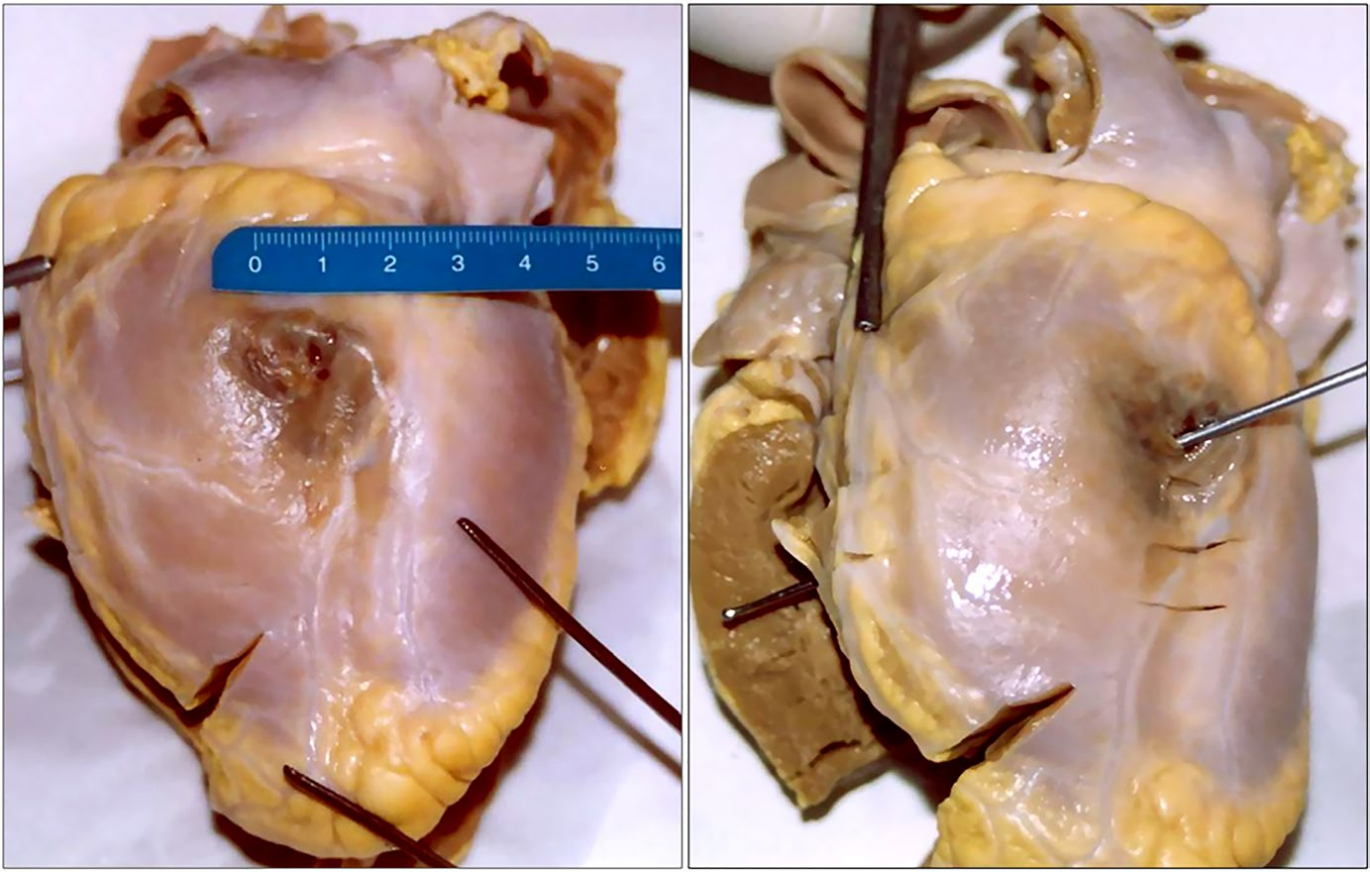
Macroscopic view of the left ventricular posterior wall after the heart dissection: A cardiac contusion was observed, and by inserting a probe, a transmural myocardial breach was documented [Color figure can be viewed at wileyonlinelibrary.com]

**FIGURE 2:**
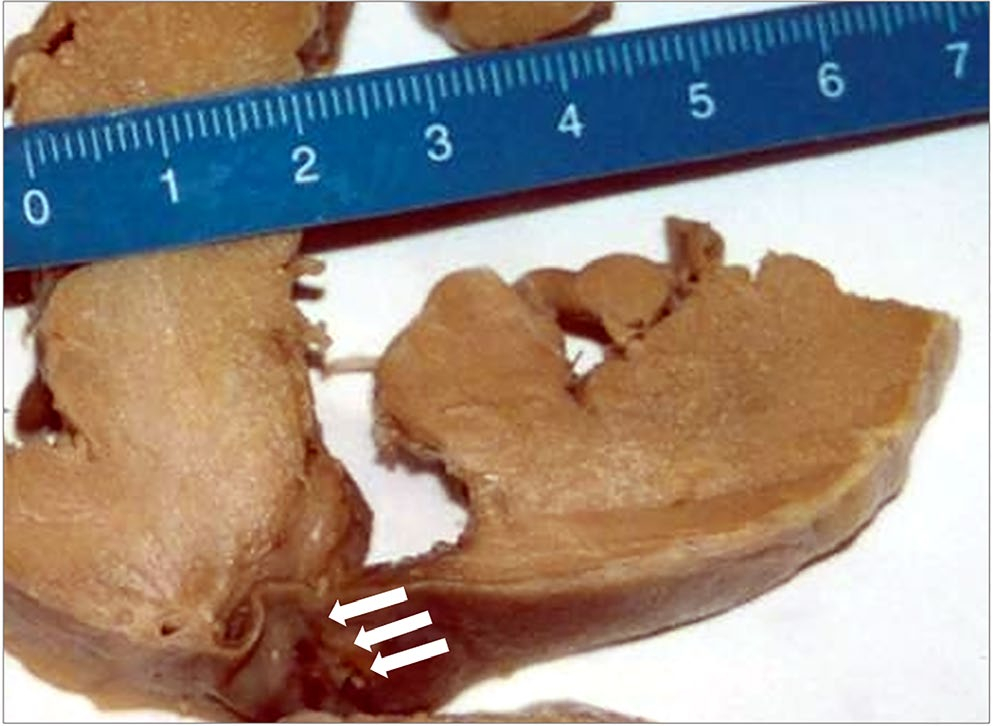
Cardiac section of the left ventricular posterior wall, with focus on the transmural myocardial rupture (arrows) [Color figure can be viewed at wileyonlinelibrary.com]

The body appeared well nourished and in a good state of preservation (weight: 53 kg; length: 177 cm). On the external examination, no relevant findings were detected. Upon dissection of the thorax, the sternum and ribs were unscathed, and the pericardial sac appeared intact, taut, and bluish in color. Inside the pericardial cavity, 700 cc of blood was documented. Most of it was fluid, and approximately 300 cc was agglomerated as a clot enveloping the entire cardiac surface. The heart, weighing 310 g, and showed moderate left ventricular hypertrophy. A rounded subepicardial hemorrhagic infiltration of 2 cm in diameter was observed in the upper third of the left ventricular posterior wall. A 3‐mm laceration with irregular margins, characterized by hemorrhagic infiltration, was detected. A transmural myocardial breach was documented by inserting a probe (Figure 1). Its margins appeared irregular, characterized by yellowish and reddish areas (Figure 2). The remaining myocardium was undamaged, and the endocardium appeared smooth and shiny with an unscathed valve apparatus. The coronary arteries were elastic, without stenosis, thrombosis, or reduced vessel lumen. Nothing relevant emerged from the dissection of all the other viscera, and at the conclusion of the gross examination, the cause of death was identified as a pericardial tamponade due to heart rupture. During the gross examination, biological fluids (blood, urine, bile, and gastric content), different samples of the viscera (brain, lungs, whole heart, kidneys, liver, and spleen), nasal swabs, and hair were collected for subsequent toxicological and histological examination.

### 2.2 Histological examination

All samples collected during autopsy underwent a post‐fixative histological examination. Samples were dehydrated using standard procedures for 20 h in an automatic histological processor with increasing ethanol solutions, clarified with xylene substitute, and embedded in paraffin with a high melting point (58–60°C). On the heart only, sampling was performed by collecting samples from the upper, middle, and lower third of both the right and left ventricles, as well as from the interventricular septum. In total, 15 cardiac samples were collected. All histological slides (3 μm thick) were prepared using a Reichert microtome and then stained with hematoxylin and eosin (H&E). Cardiac tissue was also assessed with i) Masson's trichrome staining (MT), which is specific for connective and muscle tissue; ii) phosphotungstic acid‐hematoxylin stain (PTAH) to highlight the contraction band necrosis and fibrin deposits; and iii) Perls’ Prussian blue reaction with potassium ferrocyanide (P) for hemosiderin.

**FIGURE 3:**
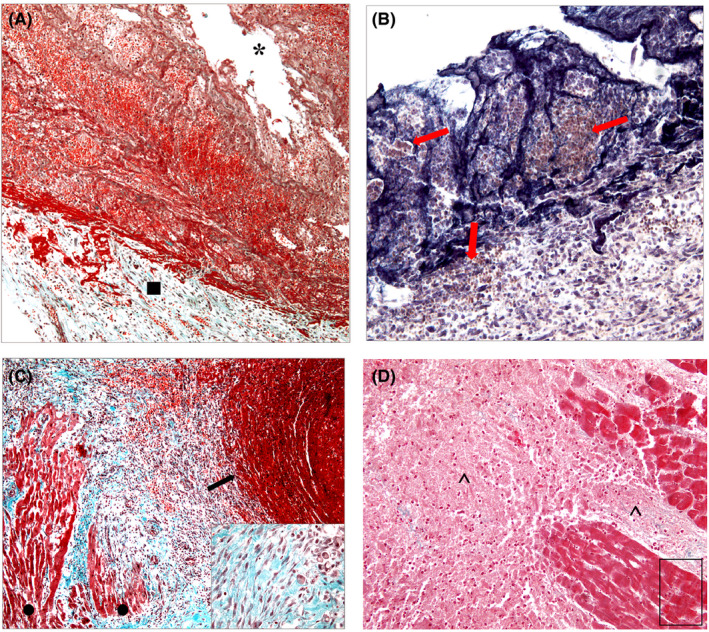
(A) Microscopic view (Masson's trichrome staining, 100×) of the myocardial breach (*) of the left ventricular posterior wall, with evidence of an acute hemorrhagic infiltration, consisting of morphologically well‐preserved red blood cells incorporated within the fibrin deposits; underneath the breach (■), a fibrous area of recent healing can be observed. (B) Microscopic view (phosphotungstic acid‐hematoxylin stain, 200×) of coarse, colored‐in‐blue fibrin strands that have incorporated several foci of morphologically well‐preserved red blood cells (arrows). (C) Microscopic view (Masson's trichrome staining, 100×) of recent healing in which there are several fibroblasts with large clearly evident ovoid nuclei, as clearly shown in the particular (bottom right corner: Masson's trichrome staining, 400×), that prove a state of proliferative activity. The smaller cells with cytoplasm and round nuclei comprise lymphocytes and macrophages. On the right side of the photomicrograph, a coarse hemorrhagic infiltration can be observed (arrow); on the left side, cardiac muscle remnants, partly incorporated in the fibrosis, are present (●). (D) Microscopic view (Masson's trichrome staining, 200×) of an extensive and well‐defined area of tissue necrosis (^), in which rare residual cellular nuclei and several foci of myocardial muscle in an advanced stage of necrosis can be observed; in the highlighted box, some contraction band necrosis are present [Color figure can be viewed at wileyonlinelibrary.com]

Histological examination (Figure 3A–D) revealed a full‐thickness myocardial rupture in the upper third of the left ventricular posterior wall, in the context of a well‐defined necrotic area. The rupture was associated with foci of acute hemorrhagic infiltration, consisting of morphologically well‐preserved red blood cells (Figure 3A) and fibrin deposits (Figure 3B), in the context of an extensive area of recent fibrosis. Numerous mature lymphocytes, macrophages, hemosiderin, and fibroblasts were also observed (Figure 3C). Moreover, neutrophilic granulocytes, as well as necrosis of myocardiocytes and multiple foci of contraction band necrosis, even at a short distance from the rupture, were detected (Figure 3D). No pathological findings were observed in the remaining myocardium or coronary arteries.

### 2.3 Toxicological analysis

Toxicological analyses were performed at the Laboratory of Forensic Toxicology of the University of Milan. The samples were tested to identify the presence of illicit drugs, alcohol, or other substances with pharmacological activity. All investigations revealed negative results.

## 3 DISCUSSION

Several factors, such as the force applied to the chest, compliance of the chest wall, and the exact timing of the application of the force during the cardiac cycle, can affect the spectrum of blunt cardiac injuries (BCI). The main consequences are myocardial contusions of different severities; arrhythmias and conduction disturbances; *commotio cordis*; injury to the major blood vessels, pericardium, or coronary arteries (usually the anterior descending artery); as well as post‐traumatic myocardial infarction and valvular or cardiac rupture [8]. The latter, whose incidence is reported to be between 0.1% and 0.5%, is the most serious and fatal uncommon complication [9], which may occur in a wide range of time periods, from a few hours to several days after a BCI [10]. The typical sites of cardiac rupture after BCI that have been reported in clinical studies are different from those that have been described in autopsy case series [11]: The right atrium and the right ventricle involvement are more frequently reported in clinical studies (8%–65% and 17–32%, respectively), compared to the left atrium and the left ventricle, whose rupture is almost only documented in autopsy case series (8–15% and 0–31, respectively) because it usually results in a precipitous death [12]. Septal rupture is an uncommon event [13], and laceration of the pericardium is even rarer, which may lead to cardiac evisceration and torsion of the major blood vessels connected to the heart.

Four main mechanisms have been suggested to explain the onset of cardiac rupture following blunt trauma: i) direct blow to the chest—the most common mechanism, with the transmission of a kinetic force that compresses the heart between the sternum and the spine [14]; ii) rapid increase in intrathoracic hydrostatic pressure due to the compression of the lower extremities and abdomen [15] with the transmission of the raised venous pressure directly to the atria and cardiac injury—a phenomenon known as the “Hydraulic Ram Effect” [16]; iii) rapid deceleration/acceleration/rotation with resultant disruption of the atria from their junctions with the vena cava and pulmonary veins [17]; and iv) severe changes in atmospheric pressure surrounding the body, as commonly seen in victims of explosion [18]. The direct blow to the chest can cause common and apparently trivial cardiac contusions, which may evolve into more or less extensive areas of myocardial necrosis due to the necrosis of myocardiocytes [19]. Such necrotic areas, which are notoriously more extensive in young patients due to the absence of collateral circulation, typically have well‐defined edges and no correspondence to the territory supplied by a particular coronary artery [19]. Exceptionally, they may cause thinning of the myocardium and evolve into a full‐thickness cardiac rupture [20].

In our case, the traumatic event that occurred may be attributed to the first typology, which is a direct blow to the chest. Indeed, it was a motorcyclist who lost control of his motor vehicle and fell to the ground, and his chest collided against one of the speed twist throttles. Blunt chest trauma could have caused the compression of the heart between the sternum and spine, potentially resulting in a BCI. The traumatic lesions found in the autopsy included one cardiac contusion in the upper third of the left ventricular posterior wall, associated with a full‐thickness myocardial rupture and massive hemopericardium. No bone fractures, pericardial tears, or injury to the intrapericardial portion of the major vessel was observed. The histologic appearance of the contusion, with organizing reaction and fibrosis around the hemorrhagic and necrotic myocardium, was consistent with a myocardial injury which occurred at the time of the blunt chest trauma 5 days previously and was not related to the resuscitation efforts. The large hemopericardium (700 ccs) was likely due to a combination of acute hemopericardium from the cardiac rupture, and a more chronic accumulation of reactive pericardial effusion (probably bloody), in response to the organizing reaction to the cardiac contusion. From a histological point of view, pronounced foci of hemorrhagic infiltration, fibrin deposits, neutrophilic granulocytes, and macrophages, as well as well‐defined necrotic areas involving myocardiocytes and multiple foci of contraction band necrosis, were detected in the extensive area of recent fibrosis. These findings were suggestive of myocardial necrosis, whose etiology could have been attributed to a necrotic evolution of a cardiac contusion, as well as to an ischemic necrosis following an acute post‐traumatic myocardial infarction. Although the morphology of a cardiac contusion resembles that of acute myocardial infarction, some useful characteristics to differentiate them have been reported in the literature. In particular, the amount of hemorrhage is more prominent in the case of contusion; moreover, in contusion, there is an abrupt change between the normal and abnormal myocardium, whereas the changes related to infarction are more gradual [20]. However, cardiac contusions ultimately heal by scar formation in a manner very similar to myocardial infarction, and once this has occurred, differentiation on purely histological grounds may be impossible [19]. Under these circumstances, reliance must be placed on the condition of the coronary arteries. In our case, the healing process was already fairly advanced, with the presence of fibroblasts and recent fibrosis. However, overall, the observed histological findings were more suggestive of myocardial necrosis as a consequence of cardiac contusion. This assumption was further confirmed by the macroscopic and microscopic absence of coronary thrombosis or other coronary abnormality. Based on these reasons, we excluded a post‐traumatic myocardial infarction, concluding that a cardiac contusion caused myocardial necrosis and cardiac rupture. The cause of the contusion was a fall from the moped and blunt chest trauma suffered by the boy a few days earlier. Although the chest trauma was anterior, a macroscopic cardiac contusion was found in the posterior wall of the heart during autopsy. This could be the result of the impact of the heart on the vertebral column. There is some evidence in the literature that the absence of rib fractures, which is a common feature in young people and was seen in our case, may be associated with more severe cardiac injury in cases of chest trauma [19]. Because the energy of the force is not dissipated by fracturing the sternum or ribs, almost the entire amount of kinetic energy is delivered to the heart [20].

The identification of the cause of death and its relationship to the blunt chest trauma raised issues of medical malpractice as the young boy was admitted and assessed at the hospital immediately after the road accident. The boy appeared to be in a good medical condition, without either bruises or rib fractures, only complained of widespread thoracic pain on palpation, and ECG revealed a normal heart rate and a LBBB. He was kept overnight for observation, and the following morning, he was discharged in a good condition. Both early‐ and late‐onset electrocardiographic abnormalities following blunt chest injuries are common; indeed, they are reported in 40% to 83% of such cases [14]. Among them, right bundle branch blocks are the most common [8], even if LBBBs may also occasionally be observed [21]. They are usually transient and of no clinical significance, although they can sometimes evolve into hemiblocks and, less commonly, third‐degree heart block [14]. To date, the diagnosis of BCI remains challenging and should be considered in all patients with significant blunt chest trauma [18, 22]. Indeed, subjects who suffer from BCI may not show any symptoms, have no severe sequelae, and may proceed toward a quick recovery [23]. Discharge is recommended in such cases only after a short period of monitoring [24]. However, there are also cases in which the clinical condition appears serious from the beginning, as well as those in which the potentially lethal cardiac pathological manifestations occur hours or days after the traumatic event, in the setting of apparent good medical condition [15]. Generally, if the ECG reveals any abnormalities, the patient should be admitted for continuous ECG monitoring [2]; serial measurements of troponin levels in addition to ECG may help in the diagnostic workup [2]. In the present case, concern was raised about insufficient investigation of the pathogenesis of the documented electrocardiographic abnormalities, which might have led to the discovery of an underlying post‐traumatic myocardial injury. Two important issues therefore arose: First, blunt chest trauma caused an undiagnosed cardiac contusion, and second, the progressive myocardial necrosis resulted in a fatal heart rupture. When a significant time interval occurs between the BCT and the cardiac rupture, the causal relationship to the blunt traumatic event may be less evident [9] and not straightforward to establish [25]. In this regard, the role of forensic pathologists is extremely important. When autopsy identifies a cardiac contusion which had not been detected clinically after a blunt chest trauma event, it is important that the pathologist determine the relationship of the contusion to the cause of death, whether that be causal, contributory, or incidental. To make this assessment, evaluation of anamnestic information is critical and should be compared with histological data.

This case is notable as it shows the potential lethality of blunt chest trauma in a young boy and demonstrates the importance of not underestimating such events, even in the absence of clinically identified injuries of chest structures.
